# Glatiramer Acetate Modifies the Immune Profiles of Monocyte-Derived Dendritic Cells In Vitro Without Affecting Their Generation

**DOI:** 10.3390/ijms26073013

**Published:** 2025-03-26

**Authors:** Jelena Skuljec, Maryam Sardari, Chuanxin Su, Julia Müller-Dahlke, Vikramjeet Singh, Marija M. Janjic, Christoph Kleinschnitz, Refik Pul

**Affiliations:** 1 Department of Neurology and Center for Translational Neuro- and Behavioral Sciences (C-TNBS), University Medicine Essen, University Duisburg-Essen, 45147 Essen, Germanyrefik.pul@uk-essen.de (R.P.); 2 Department of Neurology, Hannover Medical School, 30625 Hannover, Germany; 3 Institute for Experimental Immunology and Imaging, University Medicine Essen, University of Duisburg-Essen, 45147 Essen, Germany; 4 Department of Neurobiology, Institute for Biological Research “Sinisa Stankovic”-National Institute of Republic of Serbia, University of Belgrade, 11000 Belgrade, Serbia

**Keywords:** glatiramer acetate, human monocytes, monocyte-derived dendritic cells, multiple sclerosis therapy

## Abstract

Glatiramer acetate (GA) is the first-line therapy for relapsing-remitting multiple sclerosis (MS) and is increasingly demonstrating promising therapeutic benefits in a range of other conditions. Despite its extensive use, the precise pharmacological mechanism of GA remains unclear. In addition to T and B cells, dendritic cells (DCs) and monocytes play significant roles in the neuroinflammation associated with MS, positioning them as potential initial targets for GA. Here, we investigated GA’s influence on the differentiation of human monocytes from healthy donors into monocyte-derived dendritic cells (moDCs) and assessed their activation status. Our results indicate that GA treatment does not hinder the differentiation of monocytes into moDCs or macrophages. Notably, we observed a significant increase in the expression of molecules required for antigen recognition, presentation, and co-stimulation in GA-treated moDCs. Conversely, there was a significant downregulation of CD1a, which is crucial for activating auto-aggressive T cells that respond to the lipid components of myelin. Furthermore, GA treatment resulted in an increased expression of CD68 on both CD14^+^CD16^+^ and CD14^+^CD16^−^ monocyte subsets. These in vitro findings suggest that GA treatment does not impede the generation of moDCs under inflammatory conditions; however, it may modify their functional characteristics in potentially beneficial ways. This provides a basis for future clinical studies in MS patients to elucidate its precise mode of action.

## 1. Introduction

Multiple sclerosis (MS) is the most common chronic inflammatory disease *of the central nervous system (CNS)*, characterized by demyelinating lesions, axonal degeneration, and neurological disabilities. The primary contributors to MS pathology include autoreactive T helper cells (Th1 and Th17) and B cells, along with dysfunctional regulatory mechanisms of immune tolerance [1]. Additionally, blood-derived myeloid subsets, such as monocytes, macrophages, and dendritic cells (DCs), also play a significant role, potentially influencing disease outcomes depending on their activation state [2,3].

Monocytes develop in the bone marrow from common progenitors of macrophages and DCs and can be classified into at least two subtypes based on the expression of surface markers CD14 and CD16 [4,5]. They function as patrollers in the bloodstream, removing cellular debris through phagocytosis. In response to inflammation or injury, monocytes migrate into tissues, where they may either remain as inflammatory monocytes (iMons) or differentiate into inflammatory macrophages (iMacs) or monocyte-derived DCs (moDCs) capable of migrating to draining lymph nodes [6,7].

In the context of MS, an accumulation of monocytes has been observed in the CNS, which correlates with increased clinical severity [8]. iMons serve as the primary source of phagocytes within the perivascular cuffs of acute inflammatory demyelinating lesions associated with MS, likely initiating myelin breakdown during the active phases of the disease and clearing myelin debris during periods of remission [9,10]. Additionally, there is a notable presence of moDCs in the inflamed CNS lesions and cerebrospinal fluid (CSF) of MS patients, which exhibit altered phenotypes and functions that may influence disease progression [11]. Importantly, all three myeloid subsets can present peptides and upregulate co-stimulatory molecules, thereby facilitating the differentiation of naïve T cells into effector T cells and promoting their proliferation [6].

Glatiramer acetate (GA) is a first-line disease-modifying therapy (DMT) for the treatment of relapsing-remitting MS (RRMS). This non-biological complex drug is a mixture of polypeptides, comprising four naturally occurring amino acids: L-glycine, L-lysine, L-alanine, and L-tyrosine. GA is the active ingredient in several well-established medications, including Copaxone^®^ (Teva Pharmaceuticals, Tel-Aviv Yafo, Israel), Brabio^®^ (Viatris’ Glatiramer Acetate Injection, Mylan Pharmaceuticals Inc., Canonsburg, PA, USA), Glatopa^®^ (Sandoz/Novartis, Basel, Switzerland), and Remurel^®^ (Alvogen, Morristown, NJ, USA). Extensive studies have demonstrated the efficacy of GA in reducing the annualized relapse rate, delaying disability progression, and decreasing both the number and volume of brain lesions [12,13,14,15,16].

The pharmacological mechanisms of GA and the specific peptides or epitopes that contribute to its therapeutic effects, are not yet fully understood, but several important mechanisms have been identified [17,18]. GA reduces inflammation by promoting a shift in reactive T cells toward a Th2 response, enhancing T regulatory cells, and increasing the production of anti-inflammatory cytokines while decreasing pro-inflammatory cytokines [19,20,21,22]. Additionally, GA treatment is associated with a reduction in B cell subsets and a change in their phenotype from pro-inflammatory to anti-inflammatory [23]. Furthermore, GA enhances the cytolytic activity of NK cells against dendritic cells [24,25,26].

Recent studies have highlighted the immunomodulatory effects of GA on myeloid-monocytic lineage cells, both in peripheral circulation and within the brain [27,28,29,30,31]. In patients with RRMS undergoing treatment with GA, evidence indicates that monocytes undergo a phenotypic change, resulting in enhanced phagocytic activity and the development of an anti-inflammatory “type II” phenotype [29,30,32,33,34]. Our earlier study revealed that human monocytes exposed to GA show reduced expression of CD11c and a decreased capacity to tolerate phagocyte beads coated with a CD11c ligand [32]. Considering that CD11c is a well-established marker for DCs, we hypothesized that GA promotes the differentiation of human monocytes into phagocytes while inhibiting their maturation into DCs. To investigate this, we evaluated whether GA suppresses the differentiation and/or function of antigen-presenting cells (APCs) in vitro. Additionally, we characterized the GA’s effects on the immune profiles of monocytes.

## 2. Results

### 2.1. GA Reduces CD1a Expression on moDCs

**Figure 1:**
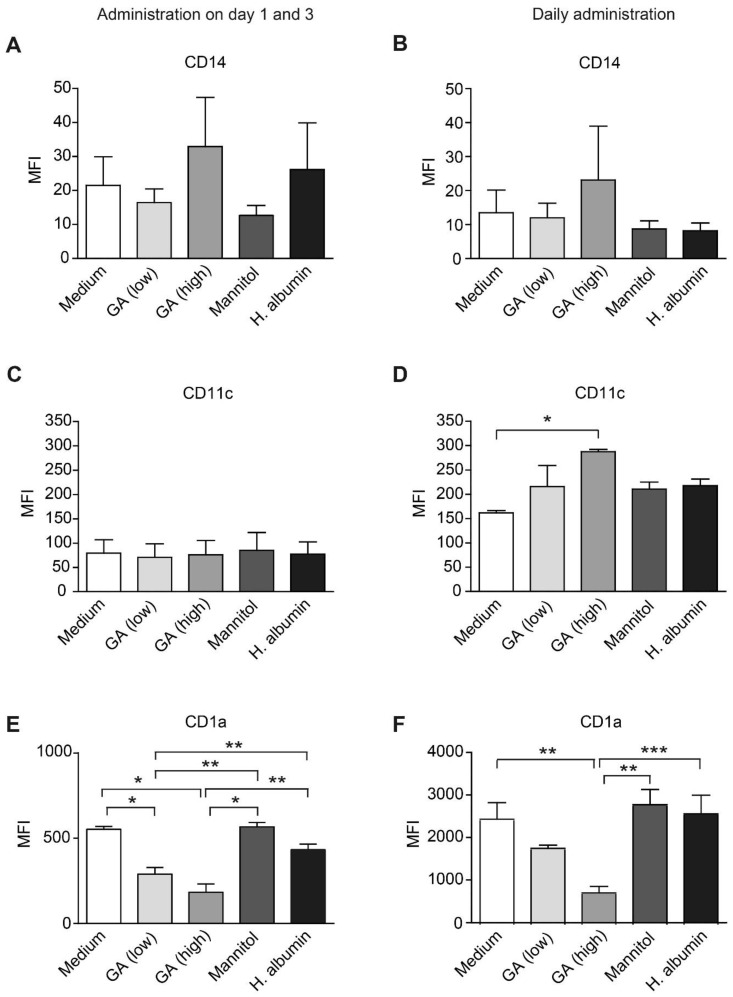
Glatiramer acetate reduces the expression of CD1a in human monocyte-derived dendritic cells. Human monocytes were obtained from the peripheral blood of healthy donors, and monocyte-derived dendritic cells (moDCs) were generated by incubating them with GM-CSF and IL-4 for five days. The moDCs were treated with glatiramer acetate (GA) added to cell cultures either on days 1 and 3 (**left**) or daily (**right**) at concentrations of 3.9 µg/mL (low) and 31.25 μg/mL (high). DMEM+ was utilized as the medium control, while mannitol (62.5 µg/mL) and human serum albumin (HA; 31.25 µg/mL) served as the vehicle and nonspecific protein control, respectively. The surface expression of CD14 (**A**,**B**), CD11c (**C**,**D**), and CD1a (**E**,**F**) on the moDCs was analyzed via flow cytometry. MFI: mean fluorescence intensity; *n* = 3 or 4; * *p* < 0.05, ** *p* < 0.01, *** *p* < 0.001 (one-way ANOVA, Tukey post hoc test).

To investigate the influence of GA on the generation of moDCs, we cultured purified human monocytes in a DC-differentiation medium for five days, adding GA at low or high concentrations, or control substances, either daily or on days 1 and 3. Notably, the concentrations used did not affect cell viability. GA did not significantly alter the expression of monocyte marker CD14 in any of the treatment regimens (Figure 1A,B). However, daily administration of GA at a higher dose (31.25 µg/mL) significantly increased the expression of CD11c on moDCs when compared to the medium control (Figure 1C,D). Additionally, GA treatment did not modify the proportion of CD14^+^ and CD11c^+^ cells (Supplementary Figure S1A–D).

Interestingly, we observed a significant decrease in the expression of CD1a under GA treatment across both treatment regimens (Figure 1E,F). Furthermore, the proportion of CD1a-positive cells significantly declined when exposed to the higher GA concentration, administered on days 1 and 3 (Supplementary Figure S1E).

### 2.2. GA Upregulates APC Activation Markers in moDCs

**Figure 2:**
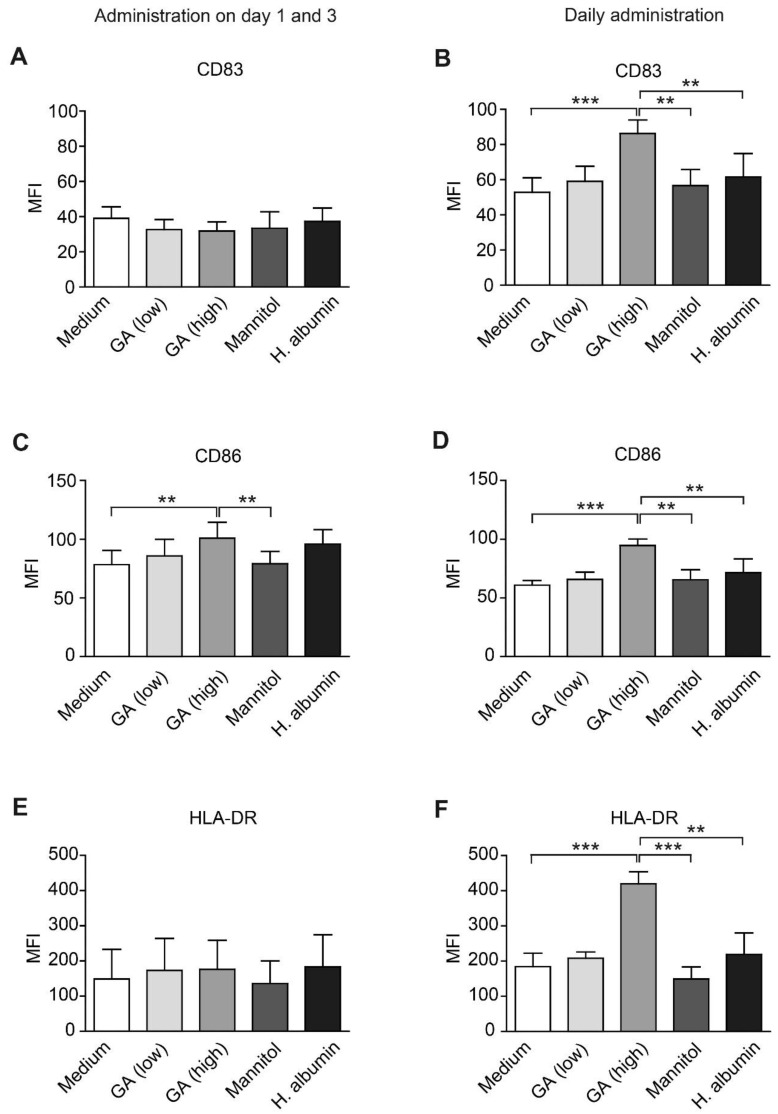
Glatiramer acetate enhances the expression of molecules involved in antigen presentation and co-stimulation on monocyte-derived dendritic cells. Human monocyte-derived dendritic cells (moDCs) were generated from monocytes isolated from the peripheral blood of healthy donors. The cells were incubated with glatiramer acetate (GA) at concentrations of 3.9 µg/mL (low) or 31.25 µg/mL (high) over a five-day period. GA was added to the cell cultures either on days 1 and 3 (left) or daily (right). DMEM+ was utilized as the medium control, while mannitol (62.5 µg/mL) and human serum albumin (HA; 31.25 µg/mL) served as the vehicle and nonspecific protein control, respectively. Surface expression levels of CD83 (**A**,**B**), CD86 (**C**,**D**), and the human leukocyte antigen (HLA)-DR (**E**,**F**) were assessed using flow cytometry. MFI: mean fluorescence intensity; *n* = 3 or 4; ** *p* < 0.01, *** *p* < 0.001 (one-way ANOVA, Tukey post hoc test).

To assess the impact of GA on the differentiation and activation of moDCs, we evaluated the expression levels of key maturation markers: CD83, CD86 and HLA-DR. Our results demonstrated that daily treatment with a high concentration of GA led to a significant increase in both the intensity of CD83 expression and the percentage of CD83^+^ cells (Figure 2B, Supplementary Figure S2B). Additionally, the expression of CD86 and HLA-DR was markedly upregulated in moDCs following daily GA administration (Figure 2D,F). An increase in the proportion of CD86^+^ cells was observed in both treatment conditions (Supplementary Figure S2C,D).

While CD68—a heavily glycosylated type I transmembrane glycoprotein—is essential within the mononuclear phagocyte lineage, our study found no effect from 5-day daily or bi-daily treatments with GA on either intracellular or extracellular levels of CD68 in moDCs. Furthermore, the proportion of CD68-positive moDCs remained unchanged across all examined GA concentrations.

### 2.3. GA Does Not Influence the Expression of Transcription Factors Essential for the Differentiation of Monocytes into Macrophages or DCs

Previous studies have indicated that a strong upregulation of the *Spi1* gene, which encodes the transcription factor PU.1, significantly inhibits the expression of the MAF bZIP transcription factor B (MafB). This inhibition promotes the differentiation of monocytes into DCs instead of monocyte-derived macrophages [35]. To assess whether GA affects the signaling pathways that regulate the direction of monocyte differentiation, we conducted gene expression analyses in the absence of DC-promoting cytokines. Our qPCR findings showed no significant changes in the expression levels of PU.1 (Supplementary Figure S3A,C) or MafB (Supplementary Figure S3B,D) in GA-treated human monocytes.

### 2.4. GA Upregulates Intracellular CD68 in Monocytes

Although we could not detect any effect of GA on the differentiation of monocytes into moDCs, we continued to investigate how GA influences the immune phenotype of monocytes.

**Figure 3:**
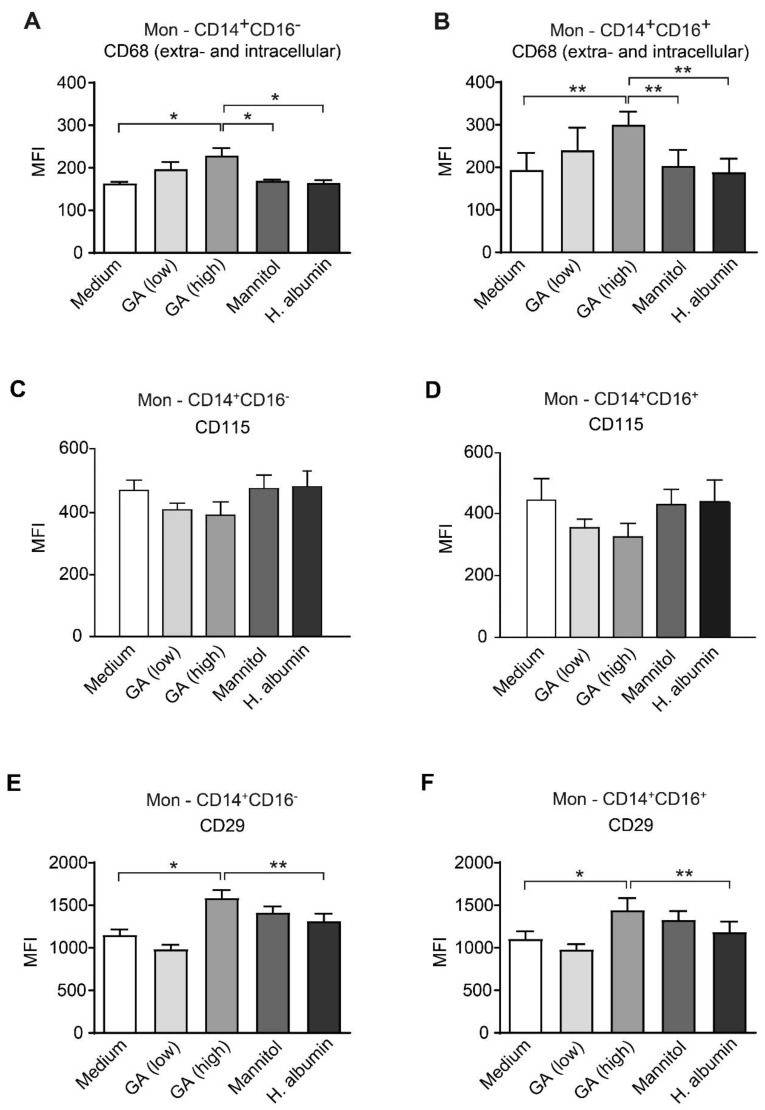
Treatment with glatiramer acetate significantly increases intracellular CD68 expression in human CD14^+^CD16^−^ and CD14^+^CD16^+^ monocytes. Human peripheral blood mononuclear cells (PBMC) were freshly isolated from healthy donors and incubated for 24 h with glatiramer acetate (GA) at low (3.9 µg/mL) or high (31.25 µg/mL) concentrations, in addition to DMEM+, mannitol (62.5 µg/mL), or human serum albumin (HA; 31.25 µg/mL) controls. The surface and intracellular expression of CD68 (**A**,**B**), as well as the surface expression of CD115 (**C**,**D**) and CD29 (**E**,**F**) on CD14^+^CD16^−^ and CD14^+^CD16^+^ monocytes were assessed using flow cytometry. MFI: mean fluorescence intensity; *n* = 4; * *p* < 0.05, ** *p* < 0.01 (one-way ANOVA, Tukey post hoc test).

We categorized the monocyte subpopulations as follows: (i) classical monocytes (CD14^+^CD16^−^)—migratory cells that infiltrate tissues and lymphoid organs, and (ii) patrolling monocytes (CD14^+^CD16^+^)—monocytes that primarily remain within the vasculature. For this analysis and the subsequent flow cytometry assessments, we utilized purified peripheral blood mononuclear cells (PBMCs). The gating strategy is outlined in Supplementary Figure S4A. Following 24 h of GA treatment, we observed no changes in the expression of surface CD68. However, both extracellular and intracellular staining of CD68 revealed a significant upregulation of this protein following treatment with 31.25 µg/mL GA in both monocyte subsets (Figure 3A,B). This finding was further supported by immunocytochemistry staining of isolated CD14^+^ monocytes (Supplementary Figure S4B). The percentage of CD68-expressing monocytes treated with GA was comparable to that of the control substances (Supplementary Figure S5A,B).

In addition, we assessed whether GA affects other important components associated with endosomal and lysosomal membranes in human monocytes, including CD63 (lysosomal integral membrane protein type-1; LIMP-1), CD107a (LAMP-1), CD107b (LAMP-2), and the scavenger receptor CD163, which serves as a marker for monocytes exhibiting anti-inflammatory properties. Our intracellular staining and flow cytometry analysis of cells treated for 24 h with 31.25 µg/mL GA revealed no changes in the expression of these proteins, both in total monocytes (Supplementary Figure S4C) and when analyzed by subpopulations.

### 2.5. Monocytes Enhance the Expression of Adhesion Molecules in Response to GA

We assessed the effect of GA on CD115, a molecule that is essential for the survival, growth, and differentiation of monocytes/macrophages. The data revealed that GA treatment did not lead to any significant changes in the expression levels of CD115 across the two monocyte subsets analyzed (Figure 3C,D and Supplementary Figure S5C,D).

Additionally, we examined the impact of GA on CD29 (integrin β1; ITGB1), a subunit of the integrin family that plays a critical role in the immune response by mediating cell adhesion and migration to inflamed tissues. Notably, treatment with 31.25 µg/mL of GA over a 24 h period significantly increased the expression intensity of CD29 on both migratory CD14+CD16− monocytes and patrolling CD14+CD16+ monocytes. This increase was clearly noticeable when comparing the treated cells to those exposed to medium and HA. However, there was no significant difference observed in comparison to the vehicle control group (Figure 3E,F). It is important to note that the percentage of CD29-expressing cells within these monocyte subgroups did not show a marked change under GA treatment when compared to the control treatments (Supplementary Figure S5E,F).

## 3. Materials and Methods

### 3.1. Isolation of Monocytes

This study was conducted in accordance with the principles outlined in the Declaration of Helsinki and received approval from the Ethics Committee at Hannover Medical School in Germany. Blood samples were collected from ten healthy volunteers, comprising both men and women, through venipuncture into tubes containing ethylenediaminetetraacetic acid (EDTA). Informed consent was obtained from all participants. PBMCs were isolated from whole blood using a continuous Biocoll separating solution (Biochrom, Berlin, Germany) with a density gradient of 1.077 g/mL. After centrifugation at 300× *g* for 25 min without break, the cells at the interface were collected and washed with phosphate-buffered saline (PBS; Biochrom, Berlin, Germany) free of Ca^2+^ and Mg^2+^. Monocytes were purified from PBMCs using magnetic CD14 MicroBeads™ (Miltenyi Biotec, Bergisch Gladbach, Germany) and MACS columns (autoMACS™ Separators, Miltenyi Biotec, Bergisch Gladbach, Germany) according to the manufacturer’s instruction. This procedure resulted in a monocyte population that was at least 90% pure, as confirmed by flow cytometry.

### 3.2. In Vitro Generation and Treatment of moDCs

MoDCs were generated as previously described [36]. Freshly isolated human monocytes were resuspended in Dulbecco’s Modified Eagle Medium (DMEM, Thermofisher Scientific, Waltham, MA, USA) supplemented with 10% fetal calf serum (FCS; Biochrom, Berlin, Germany) and penicillin-streptomycin (P/S; Thermofisher Scientific, Waltham, MA, USA), indicated as DMEM+. The cells were plated in 24-well cell culture plates (Thermofisher Scientific, Waltham, MA, USA) at a density of 5 × 10^5^ cells per well. After a 30 min long incubation, non-adherent cells were gently removed through washing, allowing the adherent monocytes to rest overnight at 37 °C in a humidified environment containing 5% CO_2_. The next day, the medium was replaced with DMEM+ supplemented with 50 ng/mL of granulocyte-macrophage colony-stimulating factor (GM-CSF) and 1000 U/mL of IL-4 (Peprotech, Inc., Cranbury, NJ, USA). The monocytes were then treated with Copaxone^®^ (Teva Pharmaceutical Industries Ltd., Petah Tikva, Israel), containing either 3.9 µg/mL or 31.25 µg/mL of GA. Along with the medium control, the following controls were included: a vehicle control containing 62.5 µg/mL of mannitol (the solvent used in the GA formulation; Merck, Darmstadt, Germany) and a nonspecific protein control containing 31.25 µg/mL of human serum albumin (HA; Baxter International, Deerfield, IL, USA). The culture medium was replaced daily or after one and three days, with measurements performed after five days in culture. This experimental design was selected to replicate clinical treatment regimens, including daily or three-times-weekly injections.

### 3.3. In Vitro Treatment of Monocytes

Freshly isolated CD14^+^ monocytes were seeded in DMEM+ at a density of 5 × 10^5^ cells per well in 24-well plates. They were allowed to adhere for 30 min in a humidified incubator at 37 °C with 5% CO_2_. Non-adherent cells were subsequently removed, and the adherent monocytes were permitted to rest overnight. The medium was then replaced with DMEM+ containing either GA at concentrations of 3.9 µg/mL, 7.81 μg/mL, or 31.25 μg/mL, or 62.5 µg/mL mannitol or 31.25 µg/mL HA. After the indicated incubation periods, the cells were detached and prepared for further measurements.

### 3.4. Measurement of Cell Viability and Apoptosis

To evaluate cell viability, cultured cells were assessed 24 h after the addition of the respective substances. Resazurin (AlamarBlue^®^; Biosource, Solingen, Germany) was diluted 1∶10 with the culture medium and incubated for 3 h at 37 °C. Optical densities were measured at 620 nm using a Tecan Sunrise spectrophotometer (Tecan, Männedorf, Switzerland). For the detection of apoptosis, an annexin V and propidium iodide (PI) dual staining kit (BD, Franklin Lakes, NJ, USA) was used, and analysis was conducted with a FACSCalibur™ flow cytometer (BD, Franklin Lakes, NJ, USA). The data were analyzed with CellQuest™ software version 3.1 (BD, Franklin Lakes, NJ, USA). Duplicate measurements were carried out in both assays, and average values were calculated from three independent experiments.

### 3.5. Immunocytochemistry

Isolated monocytes (6 × 10^4^) were seeded onto 12 mm glass coverslips placed in 4-well plates (Thermofisher Scientific, Waltham, MA, USA) and treated for 24 h with 31.25 µg/mL of GA. After treatment, the cells were fixed with 4% paraformaldehyde (Sigma-Aldrich, St. Louis, MO, USA), permeabilized with ice-cold 100% methanol, and blocked with serum to reduce non-specific binding. The monocytes were stained with a CD68-specific monoclonal antibody (Invitrogen, Carlsbad, CA, USA), followed by labelling with an Alexa Fluor^®^ 488 conjugated secondary antibody (Invitrogen, Carlsbad, CA, USA). The cells were mounted with Mowiol (Calbiochem, San Diego, CA, USA) containing 4′,6-diamidino-2-phenylindole (DAPI, Thermofisher Scientific, Waltham, MA, USA). Images were captured using a fluorescent microscope (Olympus BX61 with a camera DP72, Olympus, Tokyo, Japan).

### 3.6. Flow Cytometry

Immune phenotyping of monocytes and moDCs was performed using flow cytometry following the immunostaining of cell surface and intracellular proteins. In brief, cells were harvested from culture plates, washed and transferred to 5 mL Falcon™ round-bottom polystyrene tubes containing staining buffer (2% FCS in PBS). To block Fc receptors, the cells were pre-incubated with purified anti-CD16/CD32 antibody (Biolegend, San Diego, CA, USA) for 15 min at 4 °C. Subsequently, the cells were labeled for 30 min at 4 °C in the dark with either single or combinations of human-specific, fluorophore-labelled antibodies, diluted 1:100 in staining buffer. The antibodies included: CD14 (FITC, clone 3.9, eBioscience, San Diego, CA, USA), CD16 (APC, clone CB16, eBioscience, San Diego, CA, USA), CD11c (APC, clone B-ly6, BD, Franklin Lakes, NJ, USA), CD1a (PE, clone HI149, eBioscience, San Diego, CA, USA), CD83 (PE, clone HB15e, Biolegend, San Diego, CA, USA), CD86 (PE, clone 2331, BD, Franklin Lakes, NJ, USA), HLA-DR (APC, clone L243, Biolegend, San Diego, CA, USA), CD68 (PE, clone Y1/82A, eBioscience, San Diego, CA, USA), CD115 (PE, 12-3A3-1B10, eBioscience, San Diego, CA, USA), CD29 (PE, clone TS2/16, eBioscience, San Diego, CA, USA), CD63 (PE, clone H5C6, eBioscience, San Diego, CA, USA), CD107a (PE-Cy7, clone eBioH4A3, eBioscience, San Diego, CA, USA), and CD107b (PE, clone H4B4, eBioscience, San Diego, CA, USA). For intracellular staining of CD68, CD63, CD107a, CD107b, CD163, CD29, and CD115, an “Intracellular fixation and permeabilization buffer set” (eBioscience, San Diego, CA, USA) was utilized. Corresponding isotype control antibodies (eBioscience, San Diego, CA, USA) were used for all staining. Following washing, cytometric analyses of the stained cells were conducted using a BD FACSCalibur™ flow cytometer along with BD CellQuest™ software version 3.1.

### 3.7. Gene Expression Analysis

After culturing monocytes for 24 h with DMEM+ containing GA (at concentrations of 7.81 μg/mL or 31.25 μg/mL), mannitol (62.5 µg/mL), or HA (31.25 µg/mL), the cells were harvested and homogenized using the QIAshredder (Qiagen, Venlo, The Netherlands). RNAprotect and RNeasy Plus Mini Kit (Qiagen, Venlo, Netherlands) were used to protect the RNA integrity and isolate RNA, respectively, strictly following the manufacturer’s instructions. Subsequently, RNA concentrations and purity were assessed using the Nanodrop ND 1000 Spectrophotometer (Peqlab Biotechnologie GmbH, Erlangen, Germany), measuring A260/A280 and A260/A230 ratios. From each sample, 200 ng of RNA was reversely transcribed in a thermocycler T3000 (Analytik Jena, Jena, Germany) employing the High-Capacity cDNA Reverse Transcription Kit (Applied Biosystems, Thermofisher Scientific Waltham, MA, USA). mRNA was then amplified using TaqMan assays (Hs00162150_m1 SPIB, Hs00271378_m1 MAFβ, Hs99999905_m1 GAPDH, and Hs99999903_m1 ACTB) along with TaqMan Universal PCR Mastermix in the 7500 Fast Real-Time PCR System (Applied Biosystems, Thermofisher Scientific, Waltham, MA, USA). We improved the accuracy of our analysis by using two housekeeping genes, GAPDH and ACTB, for normalization instead of just one. For each sample, we subtracted the cycle threshold (Ct) values of these housekeeping genes from the Ct values of our genes of interest, PU.1 and Mafβ. This resulted in 2^−dCt^ values, which reflect gene expression normalized against the housekeeping genes. Next, we calculated the ratios of gene expression levels in wells treated with GA, mannitol, and HA compared to cells cultured solely in DMEM. This yielded ddCt values, which represent the difference between the dCt of the treated samples and the dCt of the medium control samples. The final results are expressed as 2^−ddCt^.

### 3.8. Statistical Analyses

Statistical analyses were performed using GraphPad Prism 9 software (GraphPad Software Inc., La Jolla, CA, USA). To assess the normality of data distributions for each assay, we applied the Shapiro–Wilk test. Following this, a one-way analysis of variance (ANOVA) was conducted, complemented by the Tukey multiple comparison test. A *p*-value of less than 0.05 was considered statistically significant. Levels of significance are indicated by asterisks: * *p* < 0.05, ** *p* < 0.01, *** *p* < 0.001, **** *p* < 0.0001.

## 4. Discussion

The exact mechanism of action of GA is still not fully understood, but its effectiveness in treating relapsing forms of RRMS is well established. Recent studies have shown that GA offers therapeutic benefits not only for RRMS but also for various neurological disorders, such as age-related macular degeneration, Alzheimer’s, Parkinson’s and Huntington’s disease, neuropsychiatric conditions and cerebral ischemia [37]. Furthermore, GA is being explored for its potential in several innovative applications, such as intratumoral drug delivery [38], combating antibiotic-resistant Pseudomonas aeruginosa [39], aiding in diabetic wound healing [40], and exhibiting antifungal properties [41]. This wide range of possible applications highlights the need for further research into GA’s mechanisms, as gaining a better understanding could lead to even more treatment options for patients with different medical conditions.

GA primarily targets leukocytes in the bloodstream and lymphatic tissues and several mechanisms have been identified to explain its immunomodulatory and neuroprotective effects [15,42]. Notably, it promotes a transition from a Th1 to a Th2 immune response, induces “bystander suppression”, and enhances the expansion of regulatory T cells (Tregs) [21,43,44,45]. Furthermore, recent studies suggest that GA may have broader effects beyond its antigen-specific action, particularly in modulating the circulatory myelomonocytic lineage [29,33,46,47]. The primary mechanisms involve the inhibition of APC activity, a shift in cytokine profiles from pro-inflammatory to anti-inflammatory [27,33,46,47], the activation of the immune checkpoint molecule VISTA (V-domain Ig suppressor of T cell activation) [48], and an enhancement of phagocytosis [32]. Our goal was to explore GA’s effects on the differentiation and immune modulation of myelomonocytic lineage cells.

In this study, we focused on human monocytes and a specific subset of DCs that develop from circulating blood monocytes during inflammatory conditions, exhibiting phenotypic markers characteristic of both monocytes and DCs [49,50]. This subset can be distinguished from conventional DCs by its unique functions and gene signatures [7,51]. MoDCs require GM-CSF for their development and play a pivotal role in initiating pathogenic responses by priming autoimmune T cells, secreting cell migration mediators, and producing IL-1β, which increases the permeability of the blood–brain barrier and exacerbates inflammatory conditions [52,53,54]. In the inflamed CNS and its draining lymph nodes, moDCs primarily present antigens, activating naïve T cells and reactivating antigen-experienced T cells, enabling them to cross the glia limitans and invade the CNS parenchyma [55,56]. In addition to their role in initiating and maintaining CNS inflammation, moDCs can also contribute to neuroprotection by restoring immune tolerance [57,58].

In our previous research, we found that treating human monocytes with GA significantly enhances their phagocytic activity. This is accompanied by a downregulation of CD11c and a reduced ability to phagocyte beads coated with a CD11c ligand [32]. CD11c (integrin αX), which is typically expressed on phagocytes along with integrin β2 [59], does not appear to facilitate phagocytosis mediated by GA [32]. Since CD11c is a key marker for distinguishing DCs from other APCs, we suggest that the GA-induced downregulation of CD11c in monocytes indicates a shift towards their differentiation into phagocytic iMon/iMac cells, rather than maintaining the moDC phenotype.

To address this, we treated human monocytes with GA along with maturation cytokines to generate moDCs. Our results showed that GA did not significantly affect the expression of CD14, which is a crucial marker for monocyte differentiation and essential for the generation of moDCs [60]. Additionally, GA did not have a notable impact on CD11c expression in moDCs compared to all control treatments. These in vitro findings suggest that GA is unlikely to interfere with the process of moDC generation.

CD1a is a transmembrane glycoprotein expressed by immature DCs, and it plays a crucial role in presenting lipid antigens to T cells [61]. Notably, CD1a^+^ moDCs are distinguished by their increased production of inflammatory mediators such as IL-12p70 and CCL1, while showing reduced secretion of IL-10 compared to their CD1a^−^ counterparts [62]. In patients with MS, CD1a^+^ moDCs can be identified in early active lesions of the brain [63]. Moreover, MoDCs derived from MS patients or exposed to their serum demonstrate a higher expression of CD1a compared to healthy controls [46,64]. This evidence suggests that CD1a^+^ DCs may be instrumental in activating auto-aggressive T cells that target lipid-rich myelin antigens, contributing to the progression of MS [11]. Our findings showed a significant reduction in both the proportion of CD1a-expressing moDCs and the overall amount of CD1a protein on the cell surface after treatment with GA in vitro. PBMCs from MS patients who received both GA and interferon-β (IFN-β) exhibited significantly lower CD1a expression compared to those treated with IFN-β alone [65]. It remains uncertain whether GA reduces the presentation of myelin components by moDCs or positively influences their secretion profile. To fully understand the implications of these findings, further research using cells isolated directly from treated patients is essential.

Our data clearly demonstrate that in vitro exposure of moDCs to GA results in a significant increase in the expression of activation and co-stimulatory markers, including CD83, CD86, and major histocompatibility complex (MHC) class II molecules (HLA-DR), indicating that these cells may actively engage in important interactions with CD4^+^ T cells. GA, as a polypeptide mixture, can bind rapidly and with high affinity to MHC-II molecules without requiring prior antigen processing, which allows it to present a diverse range of potentially active epitopes [66]. This mechanism promotes a shift in immune polarization from Th1 and Th17 towards Th2 and Tregs, while still enabling DCs to efficiently activate naïve T cells [27,67]. Research shows that GA can restore Treg function and promote the expansion of newly generated Tregs in RRMS patients, thereby helping to regulate autoimmune inflammation [20,34]. Furthermore, studies indicate that B cells treated with GA can enhance Treg development while simultaneously inhibiting the proliferation of pro-inflammatory T cells [68]. These findings highlight the importance of further investigation into GA’s role in transforming DCs from a pathogenic to an anti-inflammatory phenotype.

Transcription factors PU.1 and MafB are essential for the differentiation of myeloid progenitors into DCs and macrophages, respectively [35]. Our findings indicate that in vitro treatment with GA does not affect the expression of the transcription factors responsible for myeloid cell fate, suggesting that this drug does not interfere with the differentiation of monocytes into either moDCs or iMacs.

CD68 is used as a diagnostic marker for identifying mononuclear phagocytes, yet its specific functional role in inflammation and immunity remains unknown [69]. Primarily localized in the endosomal/lysosomal compartment, this heavily glycosylated glycoprotein can rapidly translocate to the cell surface [70]. Due to its genetic and structural similarities to other lysosomal-associated membrane protein (LAMP) family members, it has been proposed that CD68 may play a role in regulating the formation of the MHC II peptide complex and in facilitating antigen processing and presentation [69]. Our results indicate that GA treatment does not affect CD68 expression in moDCs. Nonetheless, we observed a significant increase in intracellular CD68 levels in both migratory CD14+CD16− and patrolling CD14+CD16+ monocytes. Future studies are needed to investigate the function of CD68 in both healthy individuals and patients with MS. Moreover, the expression levels of proteins involved in phagolysosome formation, polypeptide uptake, loading, and MHC II-dependent T cell stimulation—specifically LIMP-1, LAMP-1, LAMP-2 and CD163—remained unchanged in monocytes treated with GA.

The monocyte survival and differentiation marker, CD115, is associated with demyelination and immune activation during the active phase of MS and disease models [71,72]. Notably, our in vitro results showed that treatment with GA did not lead to any significant changes in the expression of this marker. Integrin beta-1 (CD29), a crucial molecule for the migration, extravasation, and recruitment of leukocytes, exhibited increased levels following GA treatment in both monocyte subsets. While our results indicate that GA does not appear to affect the survival of monocytes or their differentiation into macrophages, further research is needed to determine if this therapy influences the recruitment of monocytes to the CNS.

It is essential to recognize the limitations of this study. Notably, the cells utilized were obtained from healthy volunteers instead of individuals with MS. The difference in the immune environment could greatly influence the immune profiles of the cells we examined. Additionally, the in vitro design of the study limits the capacity to accurately model how this compound interacts with cells in the complex and dynamic milieu of the human body. Moreover, determining the concentrations of GA in cell cultures that adequately reflect the physiological conditions in treated patients is challenging, particularly given the lack of pharmacokinetic studies in MS patients. Therefore, caution is warranted when extrapolating these findings to in vivo scenarios.

In conclusion, our findings support the current concept that GA can modify the immune characteristics of myeloid-monocytic lineage cells in an inflammatory context. These data lay the groundwork for further clinical investigations in individuals with MS treated with GA, which will enhance our understanding of its precise mode of action.

## Data Availability

The original contributions presented in this study are included in the article/Supplementary Materials. Further inquiries can be directed to the corresponding author.

## Supplementary Materials

The following supporting information can be downloaded at: https://www.mdpi.com/article/10.3390/ijms26073013/s1.
