# Supplementary material for: Glatiramer Acetate Modifies the Immune Profiles of Monocyte-Derived Dendritic Cells In Vitro Without Affecting Their Generation
Source: Int J Mol Sci. 2025 Mar 26;26(7):3013. doi: 10.3390/ijms26073013 (PMC11989142; doi:10.3390/ijms26073013)
Supplement: Supplementary file 1 [file ijms-26-03013-s001.zip › ijms-3458121-supplementary.pdf]

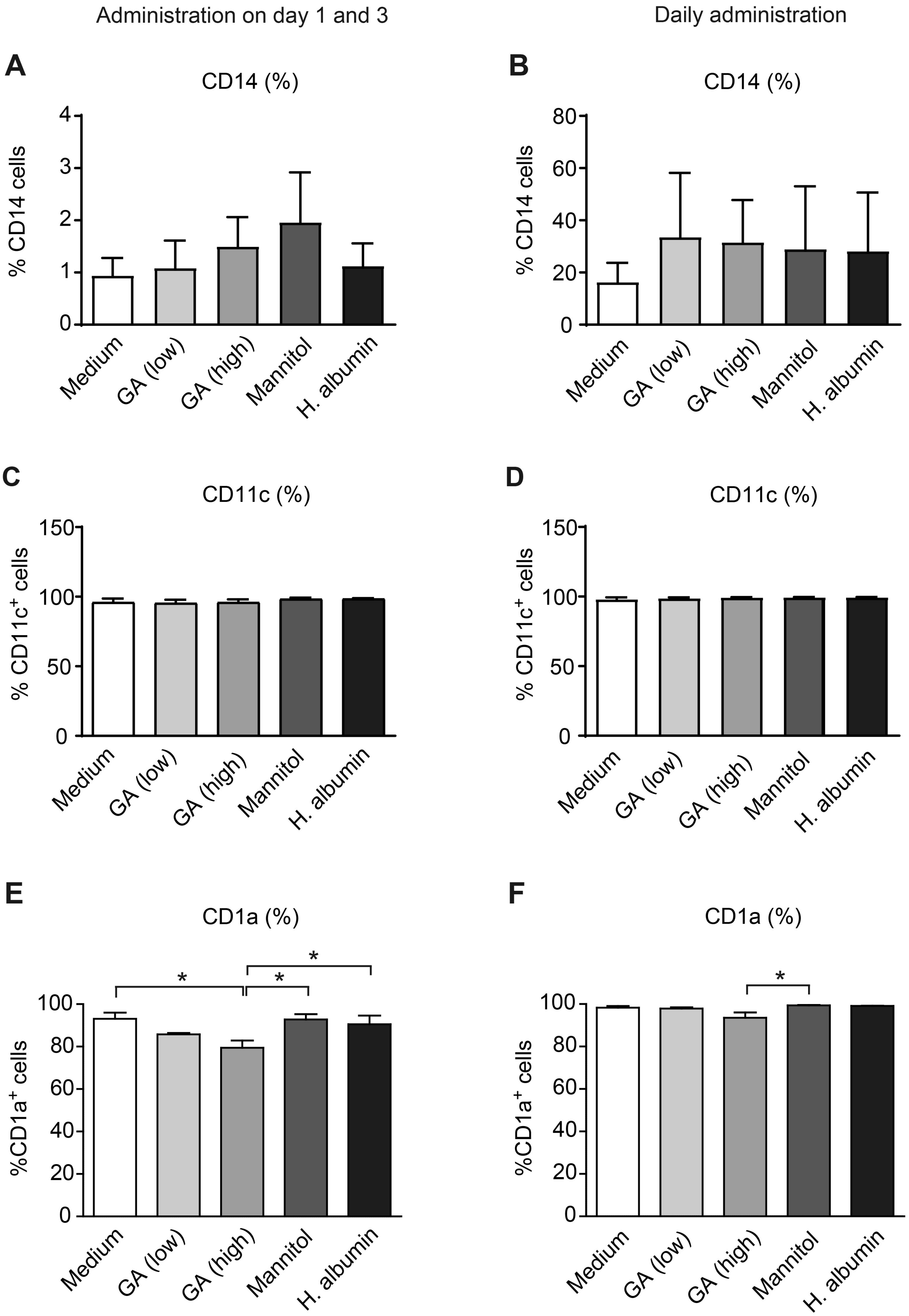

Supplementary Figure S1. Glatiramer acetate reduces the expression of CD1a in human monocyte-derived dendritic cells. Human monocytes were isolated from the peripheral blood of healthy donors and were differentiated into monocyte-derived dendritic cells (moDCs) by incubating them with GM-CSF and IL-4 for five days. The moDCs were treated with glatiramer acetate (GA) added to cell cultures either on days 1 and 3 (left panel) or daily (right panel) at concentrations of 3.9 μg/ml (low) or 31.25 μg/ml (high). DMEM, mannitol (62.5 μg/ml) and human serum albumin (HA; 31.25 μg/ml) served as the medium, vehicle, and unspecific protein control, respectively. Flow cytometry was used to assess the percentage of moDCs expressing surface markers CD14 (A, B), CD11c (C, D), and CD1a (E, F). n=3 or 4; *$p < 0.05$ (one-way ANOVA, Tukey post hoc test).

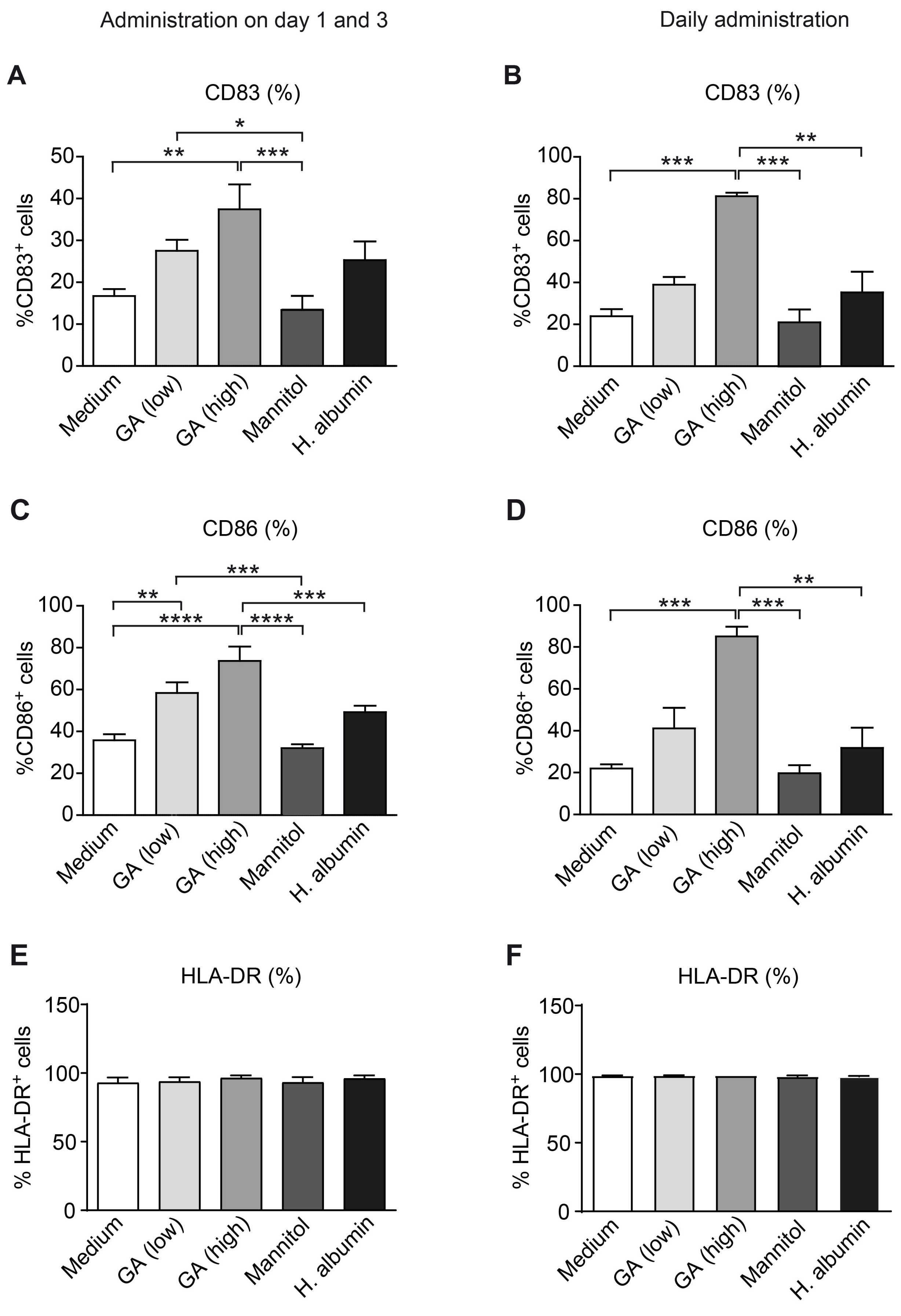

Supplementary Figure S2. Glatiramer acetate influences the expression of molecules related to antigen presentation and T cell activation in human monocyte-derived dendritic cells. Human monocytes were isolated from the peripheral blood of healthy donors and were differentiated into monocyte-derived dendritic cells (moDCs) by incubating them with GM-CSF and IL-4 for five days. The moDCs were treated with glatiramer acetate (GA) added to cell cultures either on days 1 and 3 (left panel) or daily (right panel) at concentrations of 3.9 µg/ml (low) or 31.25 µg/ml (high). DMEM, mannitol (62.5 µg/ml) and human serum albumin (HA; 31.25 µg/ml) served as the medium, vehicle, and unspecific protein control, respectively. Flow cytometry was used to assess the percentage of moDC expressing surface markers CD83 (A, B), CD86 (C, D), and HLA-DR (E, F). n=3 or 4; *$p < 0.05$ (one-way ANOVA, Tukey post hoc test).

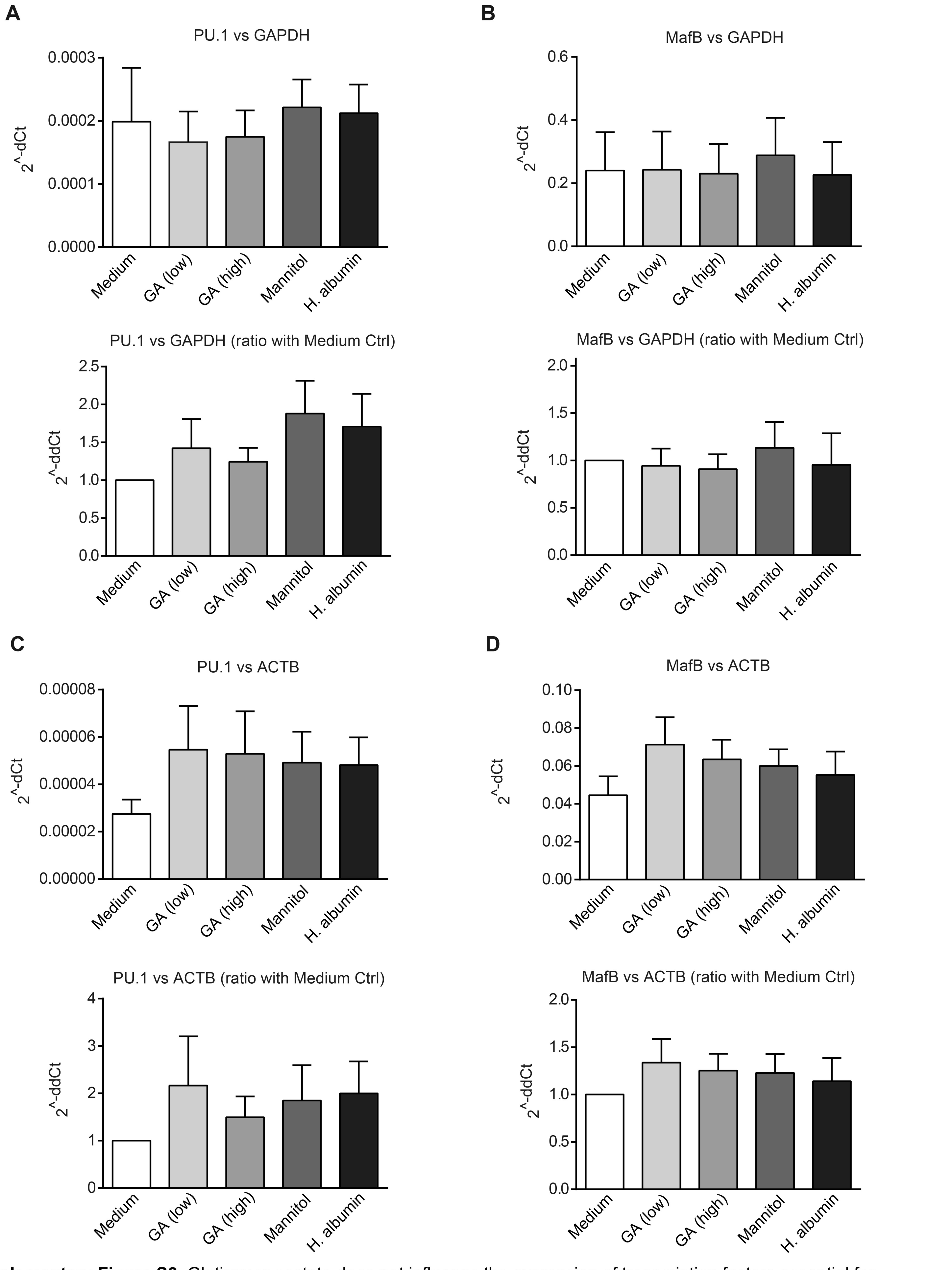

Supplementary Figure S3. Glatiramer acetate does not influence the expression of transcription factors essential for monocyte differentiation. Human monocytes were freshly isolated from peripheral blood of healthy donors and incubated for 24 h with glatiramer acetate (GA) at concentrations of 7.81 μg/ml (low) or 31.25 μg/ml (high). DMEM, mannitol (62.5 μg/ml) and human serum albumin (HA; 31.25 μg/ml) served as a medium, vehicle, and unspecific protein control, respectively. The expression levels of the transcription factors PU.1 (A, C) and MafB (B, D) were assessed using real-time PCR with TaqMan assays. Data were calculated relative to the expression of two housekeeping genes, GAPDH (A, B) and ACTB (C, D), and presented as 2^{-dCt} (normalized to the housekeeping genes) or 2^{-ddCt} (dCt values of treated cells were normalized to those incubated in the culture medium). n=8; no significant differences (one-way ANOVA, Tukey post hoc test).

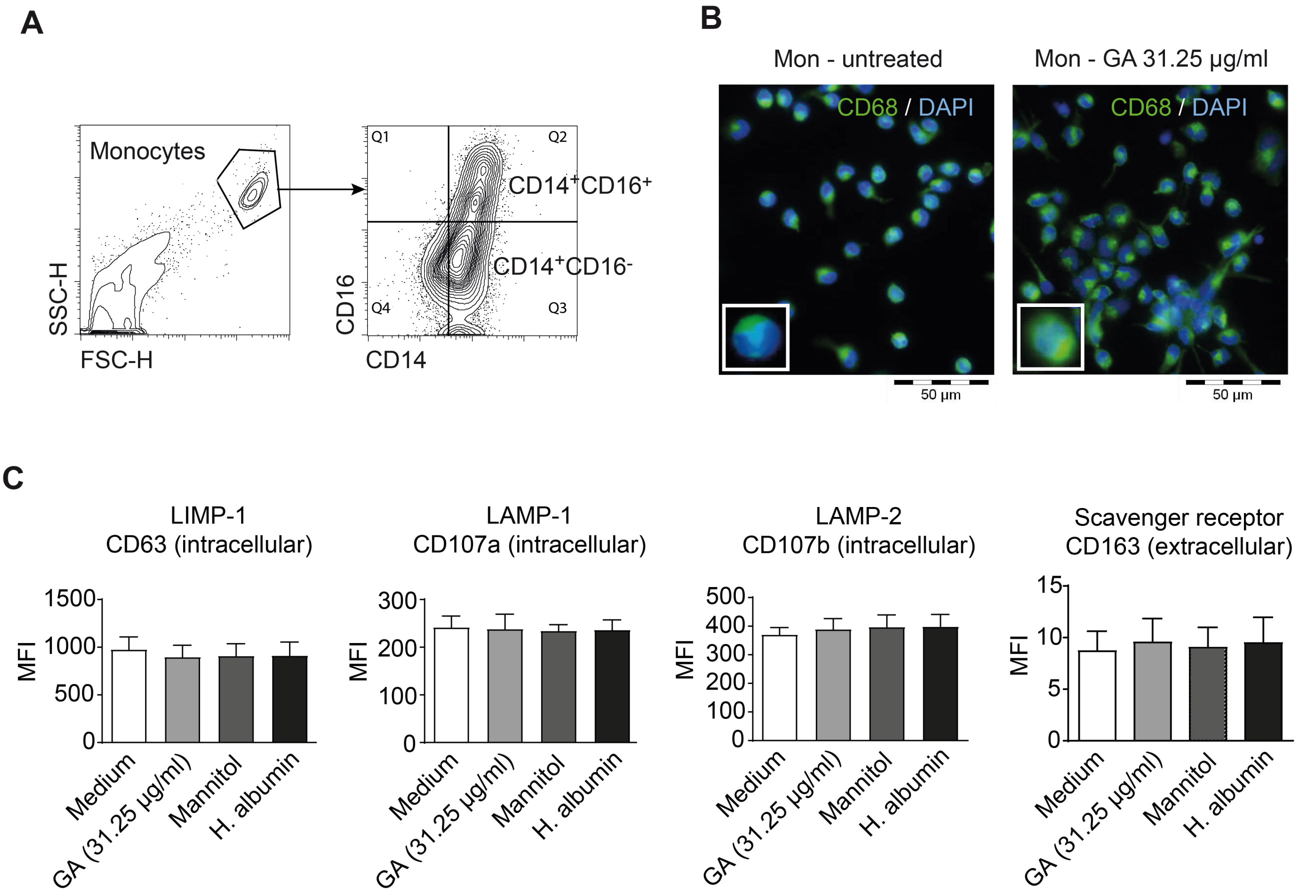

Supplementary Figure S4. Gating strategy for monocyte subsets and expression of molecules involved in the immune response. Human peripheral blood mononuclear cells (PBMCs) were obtained from healthy donors and gated based on the surface expression of CD14 and CD16 using flow cytometry (A). Fluorescent immunostaining of CD68 (Alexa Fluor® 488) was performed on isolated CD14+ monocytes treated with 31.25 µg/ml glatiramer acetate (GA) for 24 hours; scale bar = 50 µm. The cellular nuclei were labeled with 4′,6-diamidino-2-phenylindole (DAPI; B). PBMCs were cultured with GA at the concentration of 31.25 µg/ml for 24 hours. DMEM+, mannitol (62.5 µg/ml) and human serum albumin (HA; 31.25 µg/ml) were used as the medium, vehicle, and nonspecific protein controls, respectively. The mean fluorescence intensity (MFI) of proteins stained only extracellularly, or extra- and intracellularly (C) was determined using flow cytometry. n=3-4; no significant differences (one-way ANOVA, Tukey post hoc test).

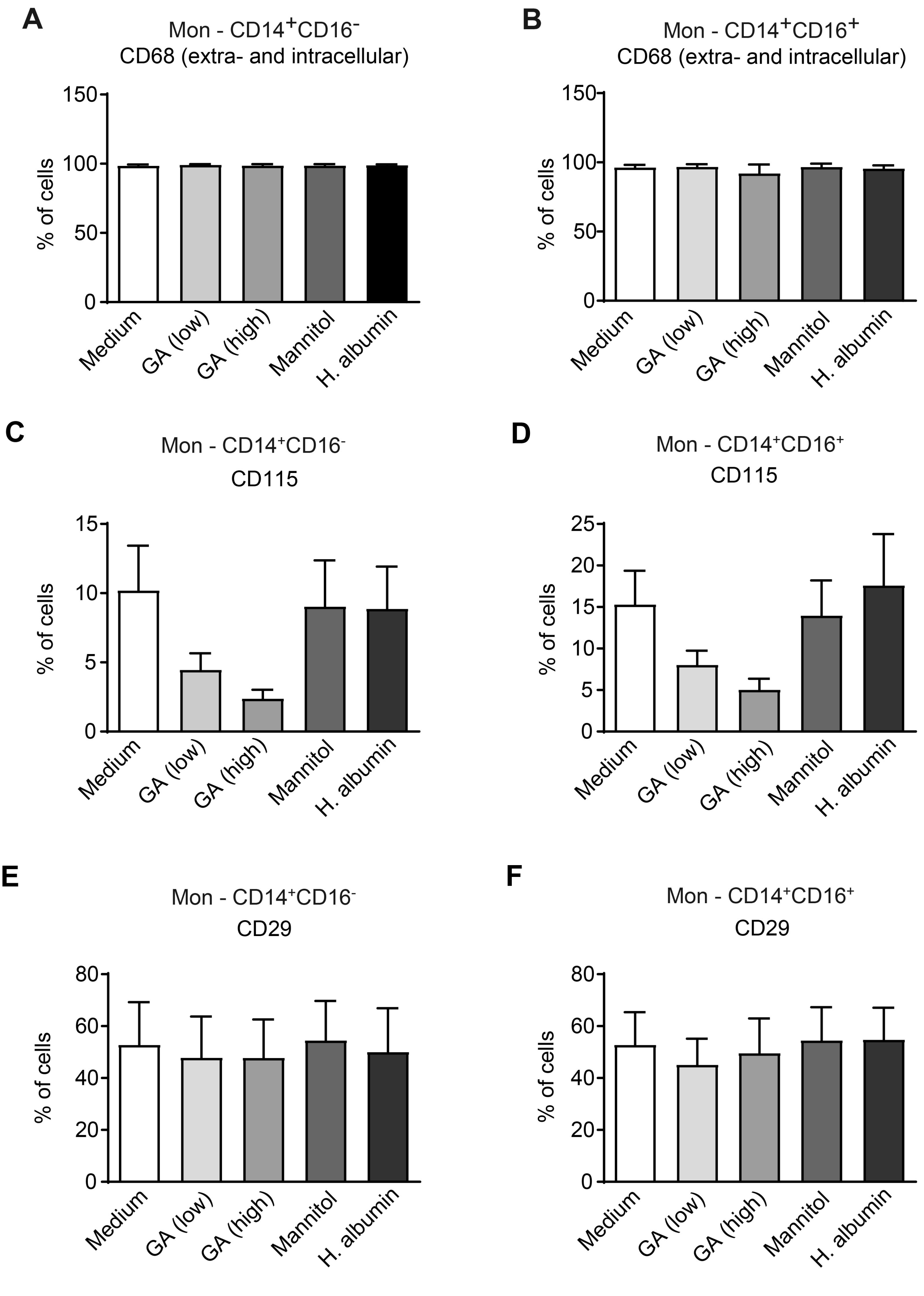

Supplementary Figure S5. The percentage of human monocyte subsets expressing CD68, CD115, and CD29 remains unchanged after incubation with glatiramer acetate. Human peripheral blood mononuclear cells were freshly isolated from healthy donors and incubated for 24 hours with glatiramer acetate (GA) at low (3.9 µg/ml) or high (31.25 µg/ml) concentrations, in addition to medium (DMEM+), mannitol (62.5 µg/ml), or human serum albumin (HA; 31.25 µg/ml) controls. The surface and intracellular expression of CD68 (A, B), as well as the surface expression of CD115 (C, D) and CD29 (E, F) on $CD14^+CD16^-$ and $CD14^+CD16^+$monocytes were assessed using flow cytometry. n=3 or 4; no significant differences (one-way ANOVA, Tukey post hoc test).
